# The Role of Climate Covariability on Crop Yields in the Conterminous United States

**DOI:** 10.1038/srep33160

**Published:** 2016-09-12

**Authors:** Guoyong Leng, Xuesong Zhang, Maoyi Huang, Ghassem R. Asrar, L. Ruby Leung

**Affiliations:** 1 Joint Global Change Research Institute, Pacific Northwest National Laboratory, College Park MD, USA; 2 Earth System Analysis and Modeling Group, Atmospheric Sciences & Global Change Division, Pacific Northwest National Laboratory, USA

## Abstract

The covariability of temperature (T), precipitation (P) and radiation (R) is an important aspect in understanding the climate influence on crop yields. Here, we analyze county-level corn and soybean yields and observed climate for the period 1983–2012 to understand how growing-season (June, July and August) mean T, P and R influence crop yields jointly and in isolation across the CONterminous United States (CONUS). Results show that nationally averaged corn and soybean yields exhibit large interannual variability of 21% and 22%, of which 35% and 32% can be significantly explained by T and P, respectively. By including R, an additional of 5% in variability can be explained for both crops. Using partial regression analyses, we find that studies that ignore the covariability among T, P, and R can substantially overestimate the sensitivity of crop yields to a single climate factor at the county scale. Further analyses indicate large spatial variation in the relative contributions of different climate variables to the variability of historical corn and soybean yields. The structure of the dominant climate factors did not change substantially over 1983–2012, confirming the robustness of the findings, which have important implications for crop yield prediction and crop model validations.

The impacts of climate change on mean crop yields have been widely recognized at the regional and global scales^1^,^2^,^3^,^4^,^5^. Climate affects crop growth and food production via complex pathways. The most direct impacts stem from the dependence of crop growth on precipitation and temperature^6^,^7^. These two variables have been recognized as the major climate factors governing crop growth regionally and globally^3^,^8^,^9^,^10^,^11^. In addition to temperature and precipitation, radiation can have considerable effects on crop growth^12^,^13^,^14^. Indeed, a response of crop yields to radiation variability can be expected because photosynthesis is primarily driven by solar radiation^15^. Moreover, solar radiation is an important driver of evapotranspiration especially in energy limited regions^16^,^17^, an essential component of the soil water balance that affects crop growth. There is some indication that the amount of solar radiation has increased by nearly 0.05 percent per decade since the late 1970s, which is equivalent to one fourth of the yearly human energy use^18^. However, the separate effects of changes in radiative fluxes on historical crop yields have not been explicitly quantified using statistical approaches.

Although the combined effects of climate factors (e.g. growing season temperature and precipitation) on crop yields are well documented in empirical studies^3^,^10^, our understanding of their isolated effects remains incomplete. Moreover, important climate variables, such as temperature, precipitation and solar radiation, interact with each other and any single variable may explain a certain fraction of the variability of the others (hereafter referred to as climate covariability)^19^,^20^, thereby complicating detection of the relationship between any single climate factor and crop yields. A recent modeling study^21^ pointed out the importance of considering surface solar radiation and its covariation with temperatures in estimating temperature impacts on crop yields, hampering the ability to quantify the relative effects of climate change on crop yields^21^,^22^. Most of the previous empirical studies adopt regression analysis without considering the effects of climate covariability, which would therefore dampen the detection of climate effects on crop yield. We hypothesize that, without excluding the effect of the covarability effects, the effect of a single climate factor on crop yields could not be accurately revealed.

Empirical models that rely on past observations of climate and crop yields offer the potential to understand historical relationships between past climate and crop yields^23^. While many studies have examined the long-term effects of climate change on crop yields, relatively a few studies have focused on the response of crop yields to inter-annual climate variability^10^. Notably, temporal fluctuation of agricultural productions can cause more devastating impacts on food price, farmers’ income and food security than long-term changes in crop yields^24^,^25^,^26^. Here, we provide a data-driven analysis to advance understanding of the effects of precipitation, temperature, and solar radiation on historical corn and soybean yield variabilities across the U.S. at the county scale. Specifically, we will address the following three scientific questions: (1) to what extent the three climate factors in combination can explain crop yields variability at the county scale over CONUS and what is the contribution of solar radiation? (2) What are the true effects of individual climate factors on the temporal variability of crop yields? Here, we perform paired analyses using different statistical techniques: one including climate covariability similar to previous studies, and the other excluding such effects. By comparing these analyses, we examine the role of climate covariability in explaining crop yield variability; and (3) Which climate variable is a more dominant climate factor in explaining the historical crop yield variations for different counties over CONUS? We focused our analyses primarily on corn and soybean in the CONUS, which account for ca. 41% and 38% of the world’s total production of these two crops, respectively. The county level analyses are expected to complement the aggregated national scale analyses, which may obscure the differences in climate influence on crop yields due to the omission of spatial heterogeneity.

## Results

### County level crop yield variability over CONUS

**Figure 1:**
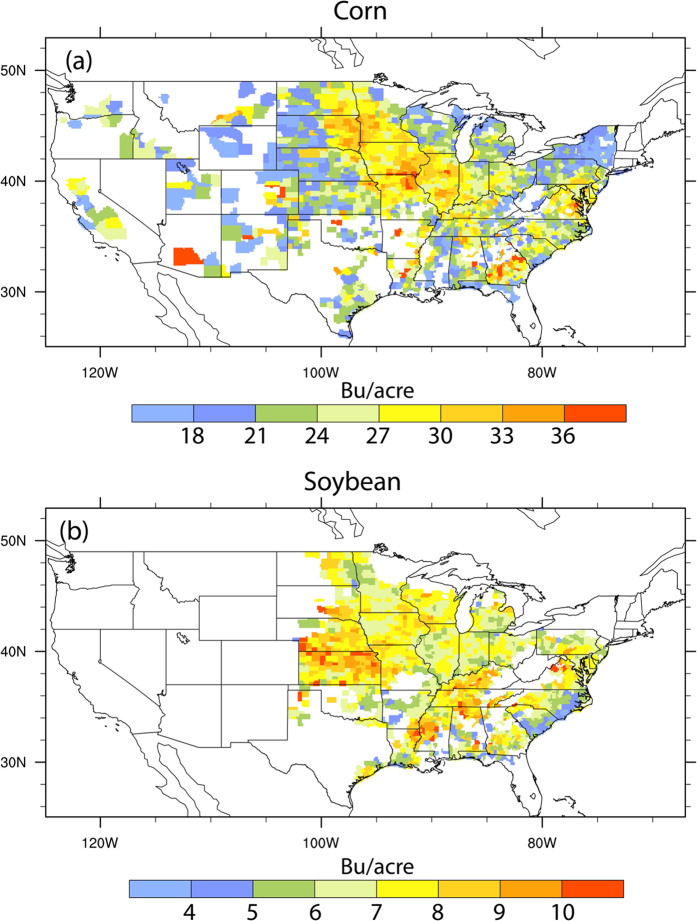
Standard Deviation (STD) of (**a**) corn and (**b**) soybean yields for 1983–2012. Figure was created by NCAR Command Language^58^.

The standard deviation (STD) of crop yields over the analysis time period is used to indicate crop yield interannual variability of corn and soybean at the county scale (Fig. 1). High STD values up to 27 Bu/acre in corn yields are found in Iowa, Illinois, Missouri, Arizona, east of South Dakota, and North Dakota while the lowest variability is obtained for Nebraska, Western Kansas and much of Western US. Although the STD is high in productive regions, the relative variability as indicated by the coefficient of variance (CV, i.e. STD normalized by the mean) is low in Iowa and Illinois (Supplementary Figure S1). For soybean yields, the highest STD up to 8 Bu/acre is distributed in productive regions such as Kansas, Nebraska, Iowa and Missouri while low STD is found in southeastern US regions. Averaged over the CONUS with the weighting based on crop-harvested area, corn yield variability (standard deviation) is 24.95 Bu/acre/year (or 21% of the average corn yields), while soybean yield variability is 7.2 Bu/acre/year (or 22% of the average soybean yields) (Table 1).

### Effects of radiation in explaining crop yield variability

**Figure 2:**
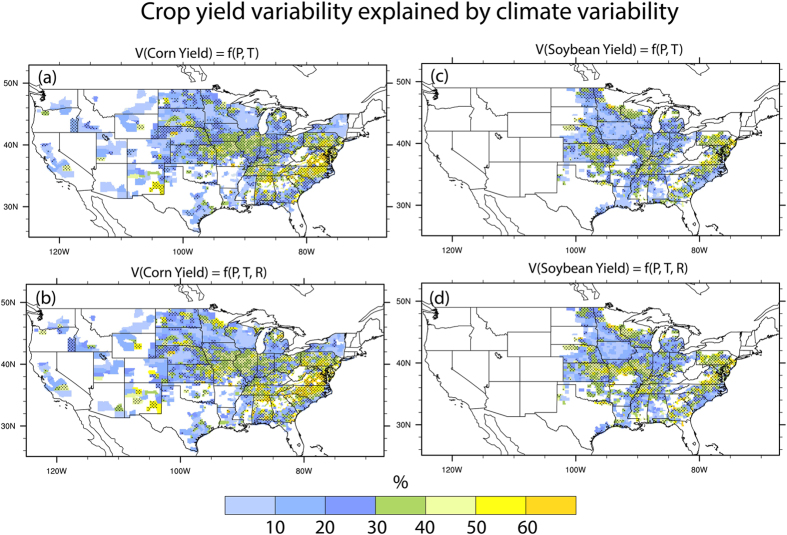
Percentage of inter-annual crop yield variability for corn and soybeans due to seasonal climate variability during 1983–2012, at the 90% statistical confidence level. (**a**,**c**) Are the crop yield variability of corn and soybean, respectively, as affected by precipitation (P) and temperature (T), while (**b**,**d**) are for the same crops but affected by P, T and radiation (R). A value of 100 implies that the entire variability in observed crop yields was explained by climate variability. Dots denote the areas where the relations between climate and crop yield is significant at the 90% confidence level. Figure was created by NCAR Command Language^58^.

During the past three decades, P exhibited the largest interannual variability over the mid-west and southeastern US (Figure S2). The largest variations of T occurred in the Midwest, especially the upper Midwest region, while Southeastern US experienced the lowest variability of T. Variability of R is high in the Southeastern US but low in the Western US. To what extent have climate variations, especially radiation changes, influenced the historical crop yield variabilities? Figure 2 shows the fraction of corn and soybean yield variability explained by T plus P. In general, climate variability has larger explanatory power over the Southeastern US (>60%) compared to other regions. In major growing regions such as the Missouri, Iowa and Illinois, up to 40% and 30% of the total year-to-year corn and soybean yield variability can be explained by T plus P, respectively. Corn or soybean yield variability in nearly half of the counties cannot be explained by climate variability at the 90% significance level, particularly in the western regions where the variability of crop yields is low. The physical mechanism behind this unexplained variability is an open question since many factors influencing crop growth and development are not explicitly considered in this study. Overall, our results indicate that 35% and 32% of the corn and soybean yield variability can be explained by T plus P (Table 2), respectively, consistent with previous findings^2^.

Notably, by factoring in R, the regression model explained more variability of both corn and soybean yields across the CONUS (Fig. 2b,d). Much of the enhanced correlation is observed over the highly productive region of US such as the Missouri, Iowa and Illinois. Averaged over the country, 40% and 37% of corn and soybean yield variability, respectively can be explained by combined effects of P, T, and R, an increase of 5% for both crops (Table 2). Overall, our results suggest that R, which was often not considered in previous empirical studies, plays a non-trivial role in influencing crop yield variability.

### Importance of climate covariability in explaining crop yield variability

**Figure 3:**
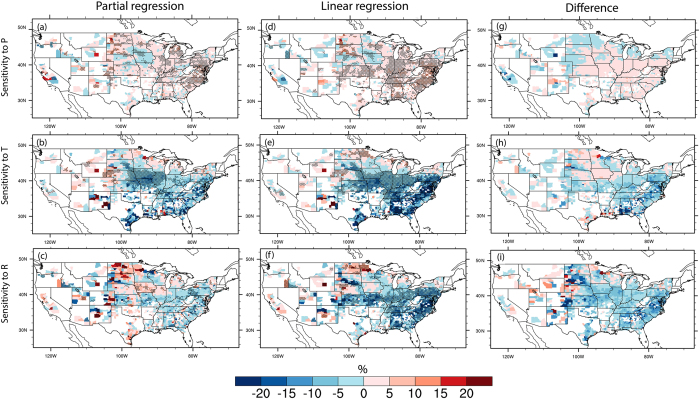
Sensitivity of corn yields to changes in precipitation (P, top panel), temperature (T, middle panel), and radiation (R, bottom panel). The sensitivity is defined as the percentage changes (%) of corn yield normalized by the long-term mean yield to each 10 mm increase of P, 1 °C increase of T and 10 W/m^2^ increase of R, respectively. (**a**–**c**) Excluded the effects of climate covariability, while (**d–f**) is derived using linear regression without excluding the effects of climate covariability. (**g–i**) Are the difference between (**b,a,d,b,f,c**), respectively. Dots denote the areas where the relations between climate and crop yield is significant at the 90% confidence level. Figure was created by NCAR Command Language^58^.

Since the climate factors as well as their impacts on crop yields can co-vary on interannual time scale, understanding the true effects of individual climate factors can help develop more effective adaptation strategies in response to anticipated climate change. Figure 3 shows the sensitivity of corn yield to P, T and R with and without excluding the effects of climate covariability. By comparing these two sets of figures, the effects of climate covariability can be examined as shown in Fig. 3g–i,. In general, P had positive impacts on corn yields over most of the corn growing regions, except for Iowa, western Nebraska, and part of Kansas (Fig. 3a). In contrast, T generally exerted negative impacts on corn yields, especially in Midwest regions (Fig. 3b). Note that many additional factors, such as local water and soil conditions as well as management practices, may complicate the task of quantifying climate influences crop growth and development. For example, the positive and negative correlations between variabilities in corn yield and T in western and eastern Nebraska are likely caused by contrasting water management practices (i.e. irrigation in the west vs. dryland in the east)^27^,^28^. By repeating our analysis for those counties with separate estimates of rainfed and irrigated yields for corn and soybean obtained from the USDA (Supplementary Figures S3 and S4), we found that the sensitivity of rainfed yields is larger in magnitude than that of irrigated yields, indicating that irrigation can reduce the dependence of crop yields on precipitation. This also illustrates the difficulty of including all potential factors affecting crop yields using statistical approaches, which have inherent limitations due to the selection of the independent variables for building the regression model. Tile drainage is used in Iowa to remove excess water^29^, consistent with the negative correlation between P and crop yield over there. In much of the western US, P has a dominant cold season peak so the growing season P should have little direct effect on corn yield, and the negative relationship is likely reflecting coincidental relationships between growing season P and corn yield with other factors such as cold season P, summer soil moisture, and water availability for irrigation.

From the partial regression, an increase of 1 K in T can lead to about 10% decrease in corn yields in the productive regions (e.g. Missouri, Iowa and Illinois). Compared to the other regions, the magnitude of corn yield sensitivity to P is larger in the water limited western US. Corn yields are expected to increase by up to 15% with a 10 W/m^2^ increase of R in some of major corn growing regions such as Iowa, North Dakota, South Dakota followed by Nebraska and California. Without excluding the effects of climate interactions, it was reported that corn and soybean yields in CONUS may decrease by 17% in response to a 1 K increase in growing season temperature^9^, which is much larger than the results obtained in this study. Indeed, comparing the results with and without excluding the compounding effects of climate interactions (Fig. 3d–f), similar response of corn yield to each climate factor is found in terms of the change direction. However, the response magnitude is much larger if the compounding effects from climate interactions are included (Fig. 3g–i).

**Figure 4:**
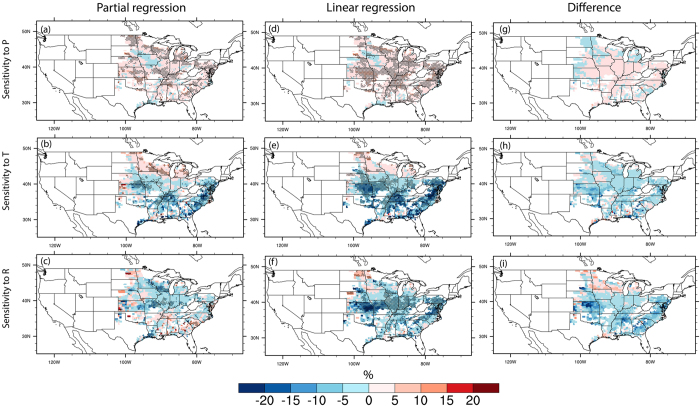
Same as Fig. 3 but for soybean yields. Figure was created by NCAR Command Language^58^.

As for soybean (Fig. 4), similar pattern of yield response to each climate factor is found. Increases of P is found to drive up soybean yields, with a magnitude comparable to corn. Soybean yields in Kansas, Tennessee and several states in the southeastern US are most sensitive to changes in T, with a 10% decrease in response to a 1 K increase in T. In Wisconsin and Illinois, soybean yields are more sensitive to R than in other regions. Importantly, the magnitude is also much larger if the effects of climate covariability are included. Overall, our results suggest that previous studies using linear regression without excluding the effects of climate covariability would overestimate the effects of single climate factor on crop yields with varying magnitude over different regions. Moreover, the crop growing counties with detected relations between climate and crop yield at the 90% confidence level would be much weaker after the effects of climate covariability is excluded. The lesser sensitivity or less significant relationship after excluding climate covariability indicates the importance of climate covariability in explaining crop yield variability. This finding is also in general consistent with the study by AghaKouchak *et al*.^30^ who showed that drought analyses based on seasonal mean precipitation substantially underestimate the risk of the 2014 California drought due to the omission of the compounding effects with temperature.

### Dominant climate factor(s) influencing historical crop yield variability

**Figure 5:**
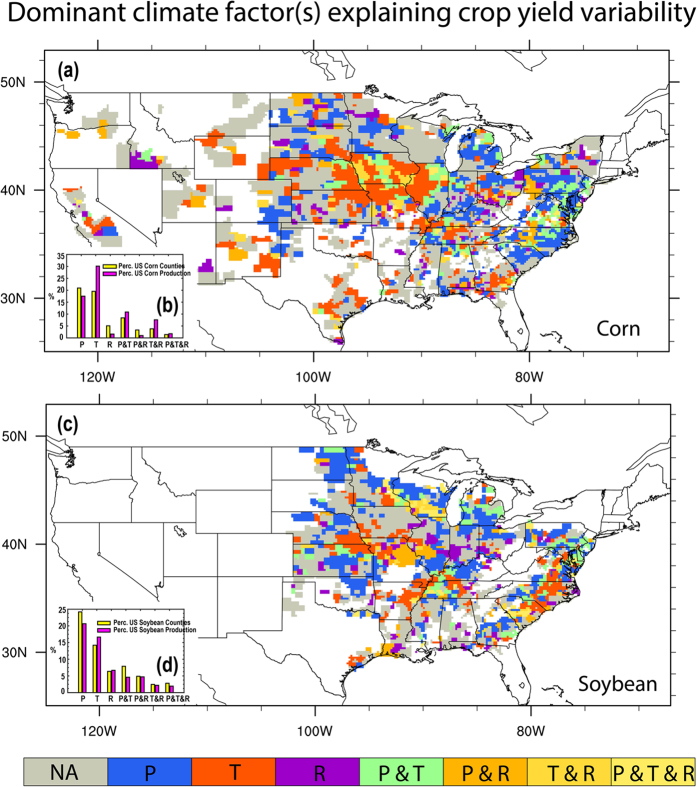
Dominant climate factor significantly explaining inter-annual (**a**) corn and (**b**) soybean yield variability during 1983–2012 at county level over US. Dominant climate factors indicate those with significant relations with crop yields at the 90% confidence level. For example, T means only T has significant relations with crop yields while T&P means both T and P have significant relations with crop yields at the 90% statistical confidence level. The Grey color indicates the counties where the crop yield variability cannot be explained by any of the single climate factor at the 90% significant level. Note the relations are obtained after excluding the effects of climate covariability. The insert bar diagrams are statistics summarizing the percentage of crop growing counties with crop yield variability dominated by each climate factor(s) and their percentage contributions to the US total productions. Figure was created by NCAR Command Language^58^.

Although many studies emphasized the importance of different climate factors, understanding of the regional differences in controls of crop yields is rather limited. Hence, a key question arises as to which climate factor is relatively more important in explaining historical crop yield variability in different counties over US? Our analyses show large spatial variation in the relative importance of different climate variables to the variability of corn and soybean yields across CONUS, as shown in Fig. 5. For corn yields, T alone was able to explain the variability in the Central Great Plains, while in the Northern Great Plains and eastern U.S., P alone accounted for most of the variability (Fig. 5a). Overall, T alone is the dominant climate factor for 20% of US corn growing counties that contributed 30% to the total US corn production (Fig. 5b). P alone also dominated corn yield variability in 20% of US corn growing counties, but they are mainly located in the Southeastern US, contributing only 17% of total corn production. The substantial spatial variation in the dominant climate factors clearly demonstrate that climate impacts are local/regional and should be addressed by considering local environmental settings. R alone only dominated corn yield variability in 5% of US corn growing counties, with a minor contribution of 2% to US total production. However, R together with T and P (i.e. R&P, R&T, and R&P&T) has affected corn yields in 14% of corn growing counties (or 12% of US total corn production).

For soybean, most of the counties, where P alone is the most important factor, are located in North Dakota, Nebraska, and Kansas, while T alone is most important in Arkansas, North Carolina and Nebraska (Fig. 5c). P alone was most important in 24% of soybean growing counties (20% of total US soybean production), while T alone was most important in 14% of the counties that contribute to a 17% of the US total soybean production (Fig. 5d). R alone was the most important factor for 6% of soybean growing counties, which produced 7% of the total US soybean production. A combination of R together with T and P, affected soybean yields in 11% of soybean growing counties (or 10% of US total soybean production). Overall, the counties where corn and soybean yield variability could be significantly explained at a 90% confidence level are located in the major corn and soybean production regions that account for 68% and 59% of the US total corn and soybean production, respectively.

**Figure 6:**
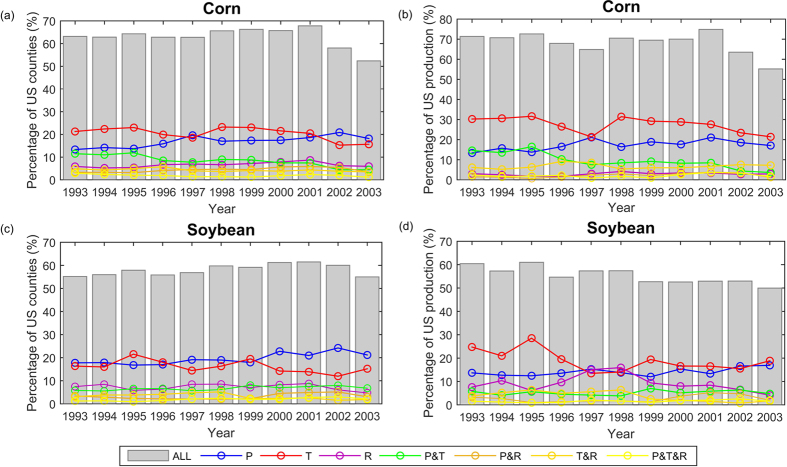
Changes in the percentage of (**a,b**) corn and (**c,d**) soybean growing counties and productions dominated by each climate factor and their combinations after applying a moving window of 20 years along 1983–2012 period. Grey bar is the total percentage explained while the solid lines represent that by each single or combination of climate factors. The x axis is the central year of the 20-year moving window, e.g. 1993 stand for a moving window from 1983 to 2002. Figure was created using software MATLAB 2015a (http://www.mathworks.com/).

How robust are the derived climate-crop yield relationships? By repeating the analyses using a 20-year window, we examined the evolutions of the dominant climate factors influencing corn and soybean yield variability (Fig. 6). For corn, T prevailed for being most important factor for both the number of counties and the share of total US production most of the time. For soybean, T was also the most influential factor in terms of the share of production while P dominated in more counties except for a period around 1995. Overall, the relative importance of the three climate factors and their combinations in influencing crop yields did not change substantially over 1983–2012, indicating the robustness of the revealed relationships.

## Discussions

There are two general approaches to explore the relationships between climate change and crop yields: numerical crop modelling^31^ and statistical modelling^23^. Due to limited availability of detailed and complete spatially resolved datasets on crop types, rotations, climate, land surface and managements as well as incomplete representation of the underlying biogeochemical processes, large spreads often exist among different crop models^4^,^32^. In this study, we adopt a statistical approach to explore the observed relations between climate and crop yields. However, statistical approach also has inherent limitations due to the selection of the independent variables for regression model and covariability between various factors.

In addition to temperature and precipitation, we considered the effects of radiation that was often omitted in previous studies. We also recognize other important climate factors such as vapor pressure deficits^33^, atmospheric CO_2_ concentration^34^, and diurnal temperature range^35^. Additionally, climate variability ranges from different scales such as intra-seasonal, inter-seasonal and inter-annual. Besides the seasonal totals, intra-seasonal climate variability can have considerable effects on crop productivity^36^. Furthermore, extreme events such as droughts, floods, heat waves may affect crop growth and productivity significantly^37^,^38^ and disproportionately^39^,^40^. Use of growing season mean climate as an explanatory variable, which is common in statistical approaches^2^,^3^,^9^,^23^,^33^,^41^, was adopted in this study, although such an approach has been criticized in that other aspects of sub-seasonal variations, such as long dry spells or heat waves, can be critical to crop growth. In addition to climate variability, crop yields can also be influenced by management practices such as conservation tillage^42^, multiple cropping^43^, Soil Mulching^44^, irrigation^45^ and fertilization^46^. Previous studies illustrated the complexity of the processes and factors influencing crop yields and highlighted the challenge of attributing crop yields changes to a subset of factors^23^. While this study addresses the covariability between climate variables and their impacts on crop yields, covariability between climate and non-climate factors could have some impacts on the results.

Without explicitly considering all of the aforementioned factors and processes, the growing season T, P, and R could only significantly explain the interannual variability in corn and soybean yields in half of the counties at a confidence level of 90%. Ray *et al*.^33^ showed that one third of crop yield variability can be explained by growing season mean temperature and precipitation. Lobell and Field^2^ found that around 30% of corn and soybean can be explained by growing season mean temperature and precipitation. Our estimates that mean growing-season climatic conditions explain ~40% and ~37% of viabilities of corn and soybean yield, respectively, is consistent with their estimates and many other relevant studies^2^,^3^,^10^. The unexplained fraction of the variability could be attributed by many other factors that are not considered as discussed above, due partially to the intrinsic limitation of statistical approaches and available data for such analyses. Aggregation of gridded climate data into the county scale add further uncertainties, as the size of most counties in the Southeast US is small compared to a 0.125 degree grid cell.

Compared to climate events induced by a single climate factor (e.g. precipitation deficits), the events with concurrent and compound extremes such as low precipitation coupled with high temperatures could be more severe and pronounced as demonstrated in the 2003 European drought^47^, 2010 Russian drought^48^ and the 2014 California drought^30^,^49^. A number of studies suggest that the chance of concurrent droughts and heat waves has increased in a warming climate and are projected to continue increasing in the near future^50^,^51^,^52^. Despite these findings, little attention has been paid to the effects of climate covariance (including concurrent extreme droughts and heat waves) on crop yields. Without excluding the effects of climate covariability, the detection of true effects of a single climate factor would be dampened. Here, we performed paired analyses using statistical approaches: one that does not exclude climate covariability similar to previous studies, and the other excludes climate covariability. By comparing the results, we investigated the effects of climate covaraibility. Furthermore, the dominant climate factors (P, T, R, or the combination of the three) are presented at the county level across the U.S. and such a pattern is found to be robust during the past 30 years. The robust pattern of dominant climate factors governing crop yield is unique in that it is obtained after excluding the effects of climate covariance, therefore is valuable for identifying effective crop management practices with the aim of alleviating negative climate change impacts on food production. The derived sensitivity of crop yields to each separate climate factor is valuable for evaluating the robustness of crop models in simulating responses of crop growth to a changing climate. This type of information is sought increasingly for adaptive measures at the national, regional and global level to ensure greater resilience in food security in a warming climate with greater likelihood of extreme climate/weather conditions that affect directly these climate variables.

## Conclusions

Understanding the historical relationship between climate variability and crop yield variability helps in gaining greater knowledge for enhancing resilience of our agricultural production systems to climate variability and change. In this study, we examined the isolated effects of temperature, precipitation and solar radiation on the county-level corn and soybean yield variability over US for the period of 1983–2012.

Our results show that temperature and precipitation combined can explain up to 32% and 35% of the year-to-year US corn and soybean yield variability, respectively. By including the solar radiation, approximately 5% more variability can be explained for these two crops. Overall, T alone is the major factor for 20% of corn growing counties, which contribute to 30% of the total US corn production. Compared to T, the number of counties affected significantly by P alone is the same, but they contribute less to the total US productions (17%), as most of them are distributed primarily over Southeastern US. For soybean, P alone affects the variability of yield over 24% of US soybean growing counties that contribute 20% total US production. These counties are located mainly in North Dakota, Nebraska and Kansas. T alone affects 14% of soybean growing regions or 17% of total US soybean production in counties located in Arkansas, North Carolina and part of Nebraska. Notably, although R alone is important for only 5% and 6% of the corn and soybean growing counties, respectively, but this effect increase to 9% and 11% under the combined effects of R, T and P, and they contribute to 10% and 17% of total US corn and soybean productions, respectively. This suggests that a relatively smaller region and number of counties in U.S. are solar energy limited an affected by R, as compared with the impact of T and P. A more detailed examination of time evolution of these relations revealed that they are relatively stable for the entire period of this study, indicating the robustness of the revealed patterns of the dominant climate factor(s) affecting crop yields. Moreover, we found that the magnitude of climate impacts on crop yields was much larger when the compounding effects among multiple climate factors was included, suggesting that previous studies that used linear regression between single climate factors and crop yields without excluding their covariance would most likely overestimate the effects of climate on crop yields.

Many studies, employing different methods, have linked climate to yields, and each has their own set of strengths and weaknesses. Complementing earlier studies that examined the empirical relations between climate and crop yield, this study provide new insights by including the effects of radiation, demonstrating the importance of climate covariability and identifying where and which climate variability has been relatively important in explaining historical crop yield variability for each county over US. If climate variability is projected to increase in the same regions where climate variability historically affected most of the crop yield variability, some strategies for stabilizing crop production in light of such variability should be developed to ensure stable future crop production and preventing future food price spikes. Hence, the county level maps developed in this study can serve as a guide (e.g. crop yields variability due to climate conditions) for agricultural policy-decisions and management practices to build greater adaptive resilience in coping with climate variability and change and their impacts on these crops. Further, the statistical models developed in this study may be used to project future yield variability together with future climate projections, and for cross-comparison the results from numerical crop models.

## Materials and Methods

### Crop yield census and observed climate data

The county-level yearly crop yields for corn and soybean from 1983–2012, along with harvest area, were obtained from the US Department of Agriculture (USDA) National Agriculture Statistics Survey’s Quick Stats database (http://www.nass.usda.gov/Quick_Stats). We calculated county-scale crop production by multiplying crop yield with harvest area. Climate data were obtained from the phase 2 of the multi-institutional North American Land Data Assimilation System (NLDAS) project^53^, which includes precipitation, air temperature and solar radiation at 0.125 grid resolutions across the conterminous U.S. The NLDAS precipitation data used observations from Climate Prediction Center (CPC) gauge data with topographical adjustment, hourly Doppler Stage II radar precipitation data, and North American Regional Reanalysis (NARR) precipitation data. The temperature and radiation are derived from the analysis fields of the NARR. The temperature was adjusted to account for the vertical difference between the NARR and NLDAS fields of terrain height. The radiation is bias-corrected against Surface Radiation Budget (SRB) dataset. The NLDAS meteorological forcing data has been widely validated and used in land surface modeling studies over the CONUS. The use of NLDAS gridded temperature, precipitation and radiation time series helps ensure the consistency of the sources of the three climate variable, as the density of weather stations recording all three climate variables are relatively non-uniform over the CONUS.

### Methodology

The annual growing season (June, July and August)^9^ mean surface air temperature (T), precipitation (P) and solar radiation (R) was calculated at each 1/8th degree grid cell and then aggregated to the county level for 1983–2012 period. The linear trend of time series of crop yields from the least squares method were first removed to screen out non-weather effects such as technological improvements^2^. Next, a multiple linear regression of de-trended crop yields onto de-trended growing season mean T, P, and R time series is developed. It should be noted that the assumption of the linear relations may not hold under all conditions due to the nonlinear response of crop growth to climate^11^,^54^. By comparing the portion of crop yield variabilitys explained by the two regression models, the added value of radiation can be quantified for answering our first scientific question.

Without excluding the effects of climate covariability, the true effects of changes in a single climate factor would be dampened. Here, we performed paired analyses using statistical approaches: one including climate covariability (with simple regression technique) similar to previous studies, and the other excluding climate covariability. In order to exclude the effects of climate covariability (the second scientific question), the partial least squares regression approach^55^ were adopted. This method has been widely used in detecting the hydro-climate variations^56^,^57^ and can isolate the effects of a single climate factor by removing statistically the effects of other controlling factors. For example, if the purpose is to understand the response of crops yields to T (target climate variable), the influence of P and R (controlling climate factors) should be removed first. Specifically, crop yield variability that can be explained by the controlling variable (i.e. P and R) are first removed by calculating residuals (r1) of regressing crop yields against the P and R. Next, the residuals (r2) of regressing the target variable (T) against the controlling variables (i.e. P and R) are computed. Finally, the linear regression of r1 and r2 calculated. The sensitivity of crop yield to the target variable is computed as the slope of the partial regression. By examining the difference between the sensitivities with and without the climate covaribility effects, the role of climate covariability in explaining crop yield variability can be explored. The statistical significance of the regressions was calculated according to the two-tailed Student’s t-test. The significance of constructed relations between climate factors and crop yield was examined for each corn and soybean growing county at a confidence level of 90%. We provided this information in a spatial map marking the dominant climate factors governing the historical crop yield variability across the US. Here, dominant climate factors indicate those with significant relations with crop yields at the 90% confidence level. For example, T means only T has significant relations with crop yields while T&P means both T and P have significant relations with crop yields at the 90% statistical confidence level. Furthermore, we repeated these procedures using a moving time window of 20 years to examine the evolution of the revealed relationships. We acknowledge the importance of other climate factors not examined here to avoid significant complexity in our analyses. However, the same analysis framework can be used by including other variables of interest and importance for other specific regions.

## Additional Information

**How to cite this article**: Leng, G. *et al*. The Role of Climate Covariability on Crop Yields in the Conterminous United States. *Sci. Rep.* **6**, 33160; doi: 10.1038/srep33160 (2016).

## Supplementary Material

Supplementary Information

## Figures and Tables

**Table 1:** The mean of coefficient of variation (CV), Standard deviation (STD) and annual crop yield over all U.S. corn and soybean growing counties for 1983–2012.

	CV	STD (Bu/acre)	Mean (Bu/acre)
Corn	0.21	24.95	117.07
Soybean	0.22	7.21	32.77

**Table 2:** The mean crop yields variability due to growing season precipitation (P), temperature (T) and radiation (R), for the entire U.S. corn and soybean growing counties, and at 90% statistical significance level.

		Percentage of Variability	Percentage of Counties	Percentage of Productions
Corn	P & T	35%	41%	63%
	P & T & R	40%	44%	68%
Soybean	P & T	32%	32%	53%
	P & T & R	37%	34%	59%

The percentage of these counties and their contributions to the total US corn and soybean production is also given.
