# Supplementary material for: The Role of Climate Covariability on Crop Yields in the Conterminous United States
Source: Sci Rep. 2016 Sep 12;6:33160. doi: 10.1038/srep33160 (PMC5018813; doi:10.1038/srep33160)
Supplement: Supplementary Information [file srep33160-s1.pdf]

The Role of Climate Covariability on Crop Yields in the Conterminous United States

Guoyong Leng[1], Xuesong Zhang[1,*], Maoyi Huang[2],

Ghassem R. Asrar[1] and L. Ruby Leung[2]

[1]Joint Global Change Research Institute, Pacific Northwest National Laboratory, College Park MD, USA

[2]Earth System Analysis and Modeling Group, Atmospheric Sciences & Global Change Division, Pacific Northwest National Laboratory, USA

*Corresponding author address: Xuesong Zhang, Joint Global Change Research Institute, Pacific Northwest National Laboratory, College Park MD, 20740.
E-mail: Xuesong.Zhang@pnnl.gov

Supplementary Figures

Figure S1 Coefficient of Variation (CV) of (a) corn and (b) soybean yields for 1983-2012.CV is the ratio of the standard deviations of crop yields over the 30-year period to the average yield over the same period. Long-term mean county-level crop production of (c) corn and (d) soybean for 1983-2012. For each year, crop yield is first multiplied by harvest area as the crop production and then the long-term mean values are calculated. Figure was created by NCAR Command Language[1].

Figure S2 Inter-annual variability (standard deviation, STD) of county-level (a) precipitation, (b) temperature and (c) radiation for 1983-2012. Figure was created by NCAR Command Language[1]

Figure S3 Sensitivity of rainfed and irrigated corn and soybean yields to one unit precipitation changes using simple regression technique (i.e. the effects of climate covariability is not excluded). Note: Very limited data exist for the separate estimates of rain-fed and irrigated crop yields over CONUS. And only those counties with data length larger than 10 years during 1983-2012 are used. Figure was created by NCAR Command Language[1]

Figure S4 Same as Figure S3 but with the effects of climate covariability excluded. Figure was created by NCAR Command Language[1]

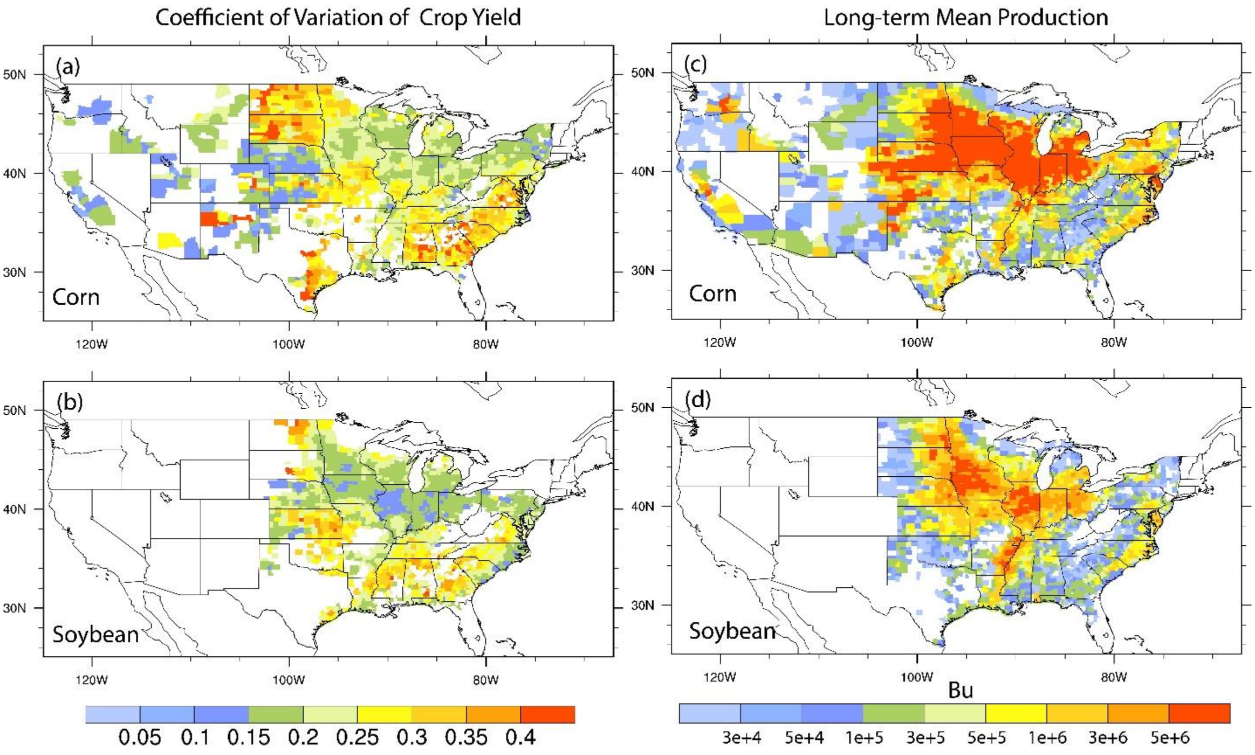

Figure S1 Coefficient of Variation (CV) of (a) corn and (b) soybean yields for 1983-2012.CV is the ratio of the standard deviations of crop yields over the 30-year period to the average yield over the same period. Long-term mean county-level crop production of (c) corn and (d) soybean for 1983-2012. For each year, crop yield is first multiplied by harvest area as the crop production and then the long-term mean values are calculated. Figure was created by NCAR Command Language[1]

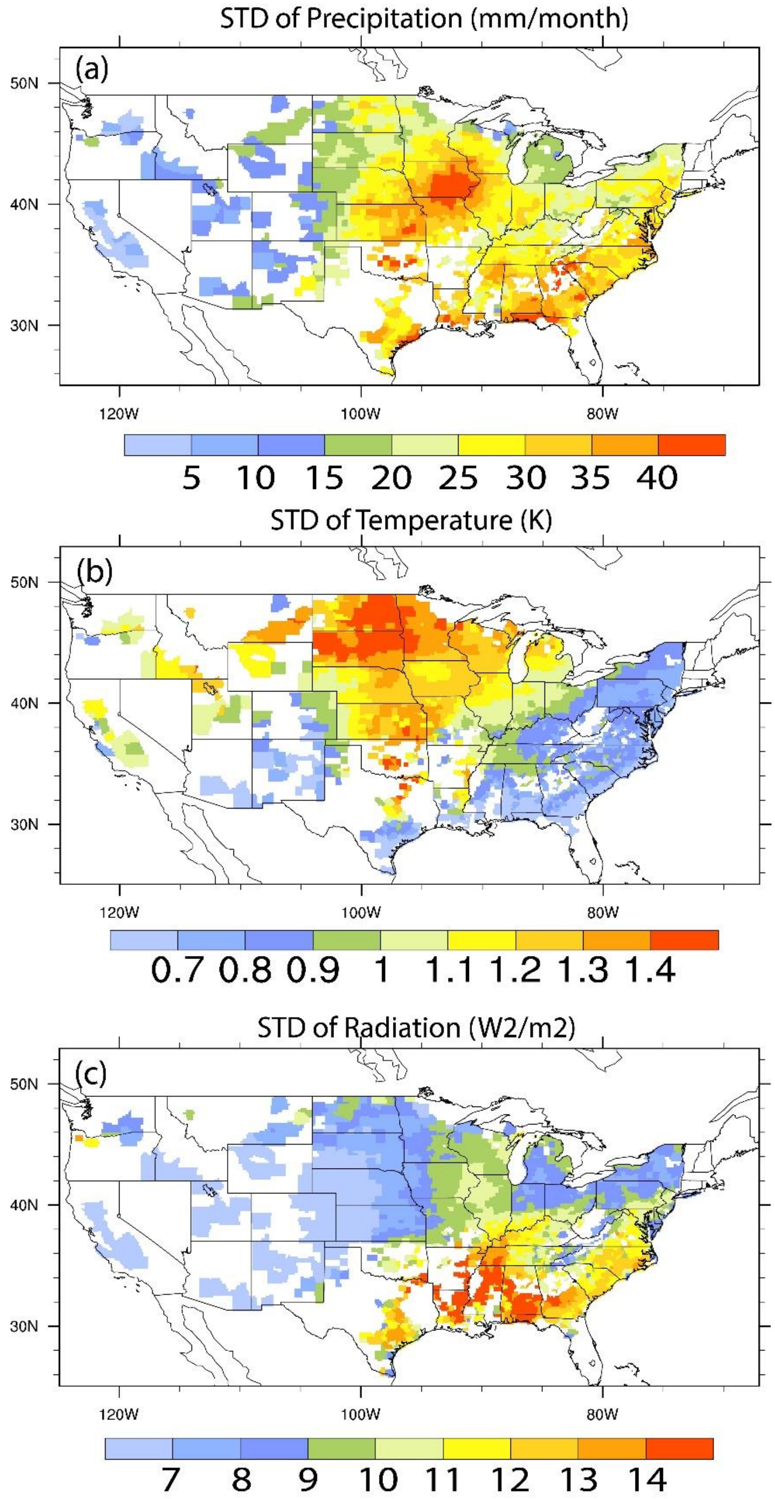

Figure S2 Inter-annual variability (standard deviation, STD) of county-level (a) precipitation, (b) temperature and (c) radiation for 1983-2012. Figure was created by NCAR Command Language[1]

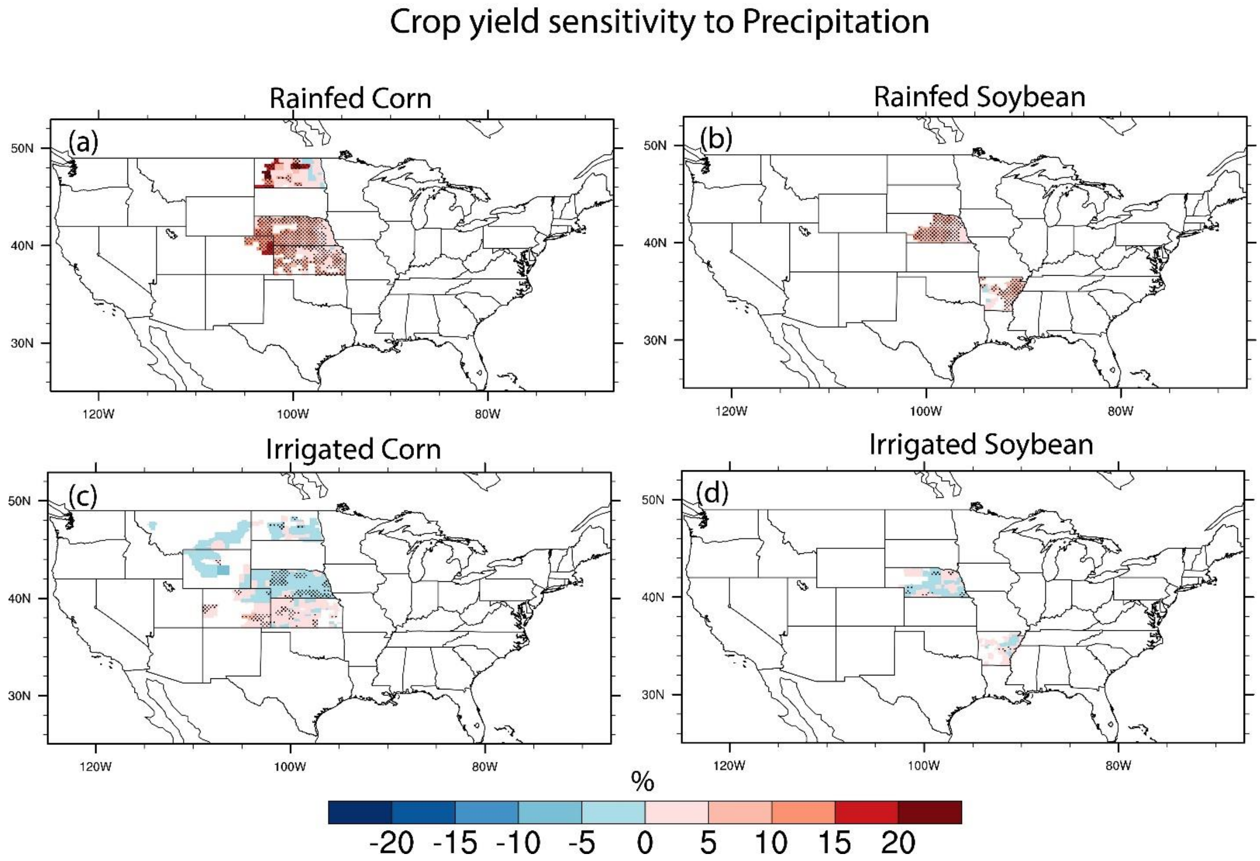

Figure S3 Sensitivity of rainfed and irrigated corn and soybean yields to one unit precipitation changes using simple regression technique (i.e. the effects of climate covariability is not excluded). Note: Very limited data exist for the separate estimates of rain-fed and irrigated crop yields over CONUS. And only those counties with data length larger than 10 years during 1983-2012 are used. Figure was created by NCAR Command Language[1]

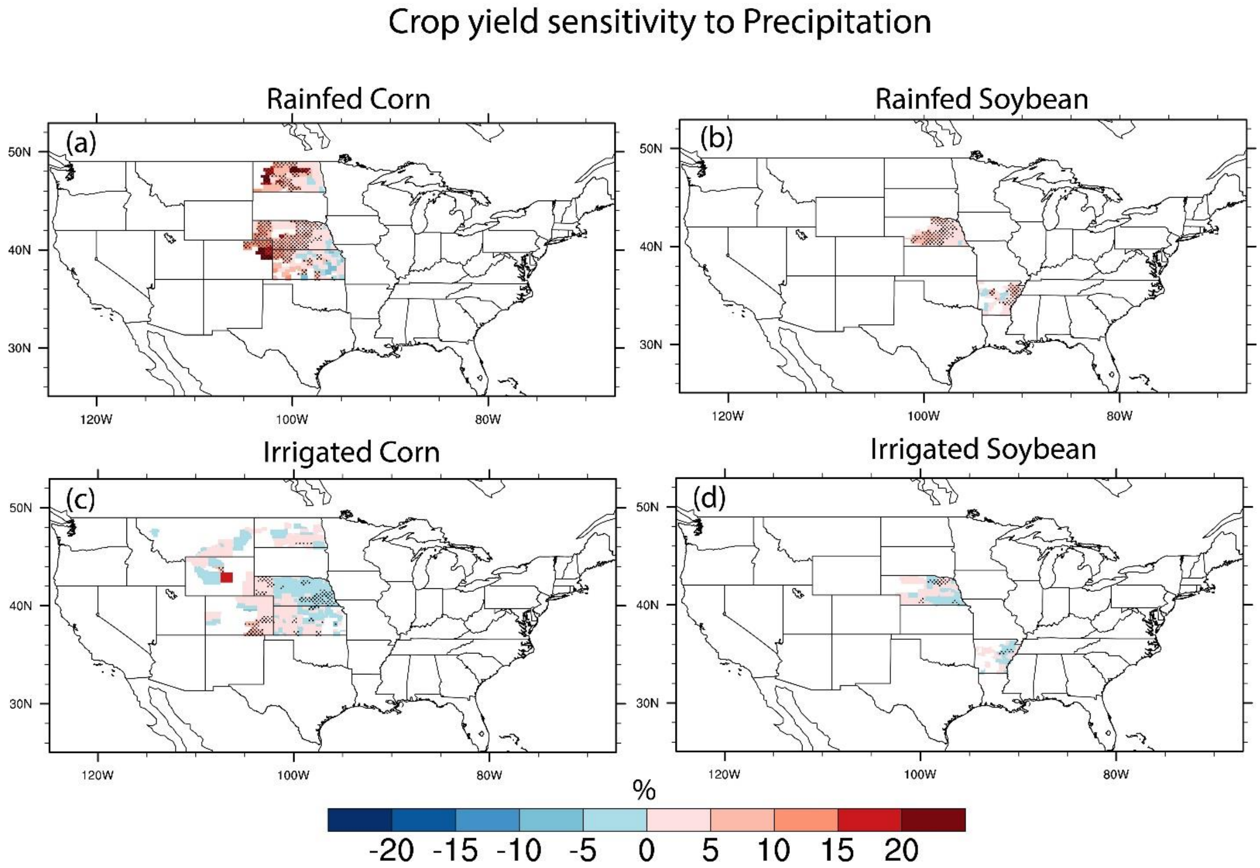

Figure S4 Same as Figure S3 but with the effects of climate covariability excluded. Figure was created by NCAR Command Language[1]

References

1. The NCAR Command Language (Version 6.1.2) [Software]. (2013). Boulder, Colorado: UCAR/NCAR/CISL/VETS. http://dx.doi.org/10.5065/D6WD3XH5.
